# The pivotal role of the intermediate fragment in initial operative treatment of olecranon fractures

**DOI:** 10.1186/1749-799X-6-9

**Published:** 2011-02-10

**Authors:** Christian von Rüden, Alexander Woltmann, Christian Hierholzer, Otmar Trentz, Volker Bühren

**Affiliations:** 1 Department of Trauma Surgery, Murnau Trauma Center, Germany; 2 Division of Trauma Surgery, University Hospital Zurich, Switzerland

## Abstract

**Background:**

In order to improve initial operative treatment of complex olecranon fractures we searched for new determining details. We assumed that the intermediate fragment plays a decisive role for anatomic restoration of the trochlear notch and consecutive outcome of initial operative treatment.

**Methods:**

80 patients operated with diagnosis of complex olecranon fracture were identified in an 8-year-period from trauma unit files at two European Level 1 Trauma Centers. Retrospective review of all operative reports and radiographs/computer-tomography scans identified patients with concomitance of an intermediate fragment. The Patient-Rated Elbow Evaluation Score was calculated for 45 of 80 patients at a minimum of 8 months postoperatively (range 8-84 months).

**Results:**

29 patients were treated with stable internal fixation with figure-of-eight tension band wire fixation and 51 patients with posterior plate osteosynthesis with/without intramedullary screw. An intermediate fragment was seen in 52 patients. In 29 of these 52 patients, the intermediate fragment was described in operative report. 24 of these 29 patients were treated with posterior plate osteosynthesis, and 5 patients with figure-of-eight tension band wiring. Complications included superficial infection (2 patients), secondary dislocation (3 patients) and heterotopic ossifications (1 patient). Functional outcome demonstrated a total PREE score of 9 points on average in 45 of 80 patients.

**Conclusion:**

An extraordinary amount of patients showed an intermediate fragment. Consideration, desimpaction and anatomic reduction of the intermediate fragment are necessary preconditions for anatomic restoration of the trochlear notch. There is no clear benefit for plating versus tension band wiring according to our data. In the operative report precise description of the fracture pattern including presence of an intermediate fragment is recommended.

## Background

Approximately 10% of fractures of the adult elbow involve the olecranon process of the proximal ulna and range from simple non-displaced fractures to complex fracture-dislocations of the elbow [1]. The proximal ulna forms a 190 degree arc around the olecranon known as the trochlear notch [2]. Articular surface incongruity of more than 2 mm leads to posttraumatic arthritis [3]. Open reduction and internal fixation is the standard treatment for displaced olecranon fractures [4]. The surgical technique is dependent on a variety of factors including patient factors, the fracture pattern, and the mechanical stability of the osteosynthesis applied to stabilize the fracture [5]. Several treatment options for open reduction and internal fixation have been described, including tension band wiring [6], plate fixation, triceps advancement after fragment excision, intramedullary locking compression nailing and intramedullary screw fixation. The so called "home run" screw provides excellent fixation of the proximal fragment into the ulna shaft [1,7-11]. Anatomic reduction and restoration of the joint surface and contour of the trochlear notch is essential for good outcome of olecranon fractures [1]. However, long-term outcome following initial surgical management of complex elbow injuries is unknown [12]. Primary elbow instability and fracture morphology are prognostic factors for elbow function and development of arthritis after operative treatment of olecranon fractures [13]. Fixation or replacement of injured bony elements, ligamentous repair, and hinged fixation may be used to successfully manage complex elbow instability [12]. There are several well-established classifications of olecranon fractures e.g. Mayo and Schatzker-Schmeling classification. Mayo classification type II and III and Schatzker-Schmeling classification type B and D describe an intermediate fragment of the trochlear notch which is frequently seen in comminuted olecranon fractures. Although the intermediate fragment is known to be critical for reconstruction and stabilization of the olecranon structure its importance is not reflected in established classifications.

As a result, fracture analysis lacks identification of the intermediate fragment in the diagnostic work up, operative reports do not describe in detail fracture pattern and presence of the intermediate fragment, and insufficient fracture reduction and unstable fixation techniques using figure-of-eight tension band wire fixation were used in many cases resulting in a high rate of revision surgery. Hypothesis of this study was that the intermediate fragment plays a key role for anatomic restoration of the trochlear notch contour and consecutive outcome of initial operative treatment of complex olecranon fractures.

## Methods

Between April 2001 and June 2009, 80 patients with diagnosis of complex olecranon fracture (Mayo classification type II and III; Schatzker-Schmeling classification type B and D) were operated in two European Level 1 trauma institutions [14-17]. 71 patients were recruited from Trauma Center Murnau data base (2001-2007, and 2009) and 9 patients from University Hospital Zurich data base (2008). Preoperative diagnostic work up, operative and post-operative treatment were the same in both hospitals. Criteria for a complex olecranon fracture include:

Comminuted

Multi-fragmentary

Dislocated

Soft tissue damage

**Figure 1:**
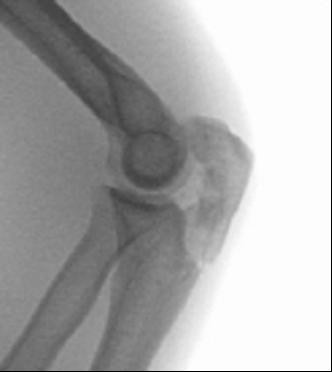
**Patient 1: Twenty-four-year-old male after bike accident**.

**Figure 2:**
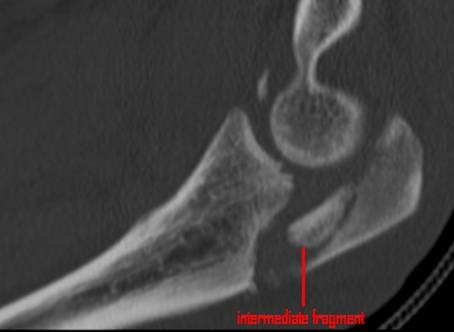
**Post-traumatic CT scan shows closed olecranon fracture classified as Mayo type IIIb fracture**.

**Figure 3:**
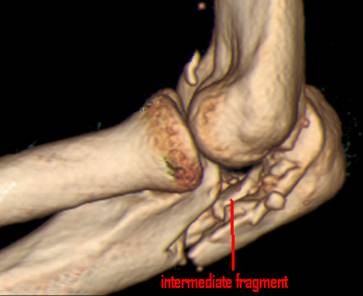
**Schatzker-Schmeling type B fracture with intermediate fragment**.

**Figure 4:**
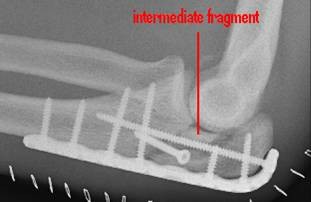
**Internal fixation with posterior plate and intramedullary "home run" screw into the ulna shaft**.

**Figure 5:**
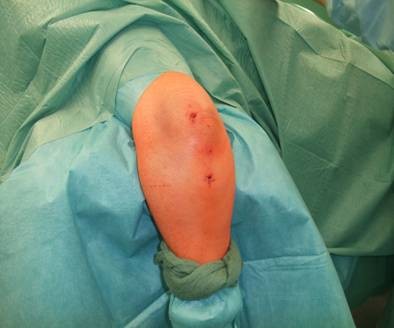
**Patient 2: 85-year-old female after fall with olecranon fracture Mayo type IIb, Schatzker type B in prone position on the operating table**. The arm is placed on an arm table.

**Figure 6:**
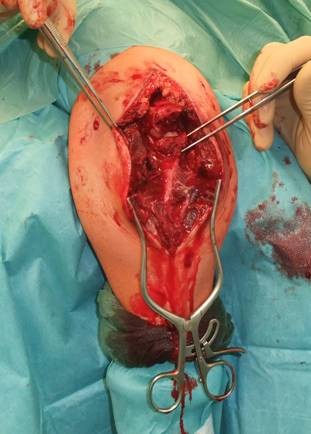
**Midline posterior approach with skin incision over the dorsal aspect of the distal humerus with a lateral curve around the radial aspect of the olecranon, and preparation of the soft tissue envelope directly to the olecranon**. Desimpaction of fracture fragments under direct view.

**Figure 7:**
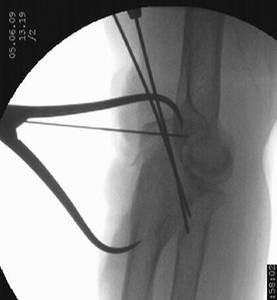
**Reduction of the intermediate fragment using a clamp between the distal and proximal partner fragment of the fracture into the interface between trochlear notch and the humerus trochlea**.

36 patients were women and 44 were men, with a mean age of 54 years (range 20-89 years, standard deviation (SD) 17.9). The average age of the 36 women was 59 years, compared with 46 years in men. 28 of these injuries were the result of a fall from a standing height and 52 were caused by a higher-energy accident, including 17 falls from a substantial height, 14 falls down stairs, 13 sports accidents and 8 motor vehicle accidents. Retrospective review of all operative reports and radiographs/computed tomography (CT) scans identified all patients whose fracture pattern demonstrated presence of an intermediate fragment [Figures 1, 2, 3]. For stable fixation traditional figure-of-eight tension band wiring or plate fixation with or without an additional intramedullary so called "home run" screw was utilized [Figure 4] dependent on fracture pattern and classification. Operative technique of common figure-of-eight tension band wire fixation is well known and not described repeatedly within this study. Osteosynthesis with plate fixation and an additional "home run" screw was performed as follows: The patient was positioned in prone position on the operating table, and the arm was placed on an additional arm table [Figure 5]. A tourniquet was applied to the upper arm. The arm was washed and draped under sterile conditions and the tourniquet was inflated. A midline posterior approach was performed with skin incision over the dorsal aspect of the distal humerus approximately 3 cm proximal to the olecranon tip with a lateral curve around the radial aspect of the olecranon [Figure 6]. Skin incision is not placed over the olecranon to avoid secondary problems with skin healing and scar formation. The deep fascia was incised in the midline and the proximal ulna and the olecranon were exposed. Following irrigation and debridement of the fracture hematoma, fracture fragments, specifically the intermediate fragment, were desimpacted under direct view. Reduction of the intermediate fragment was performed using a clamp between the distal and proximal fragment of the fracture into the interface between trochlear notch of the olecranon and the humerus trochlea [Figure 7].

**Figure 8:**
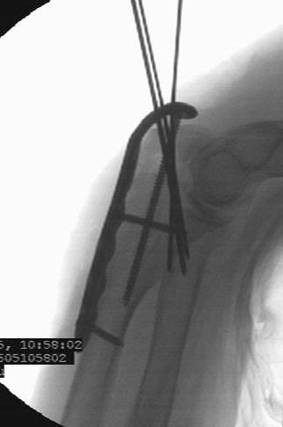
**Intermediate fragment in anatomical position on the contour of the trochlear notch, and temporary fixation of the intermediate fragment with K-wires**.

**Figure 9:**
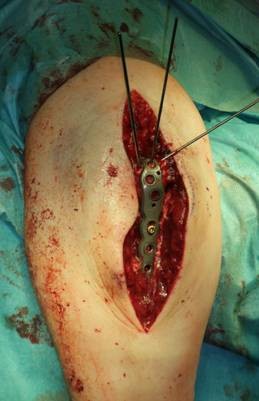
**For osteosynthesis a pre-contoured locking plate was used**.

**Figure 10:**
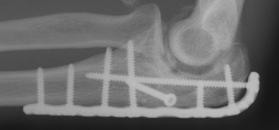
**Additional interfragmentary screw fixation of single fragments sometimes may be necessary**.

**Figure 11:**
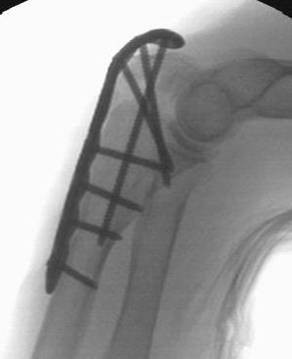
**Completed internal fixation with pre-contoured locking plate with intramedullary "home run" screw**.

**Figure 12:**
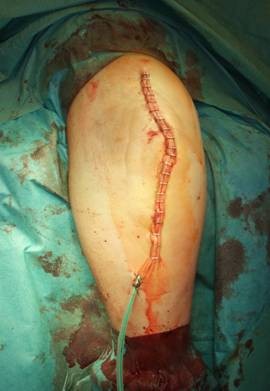
**Situation after primary wound closure**.

**Figure 13:**
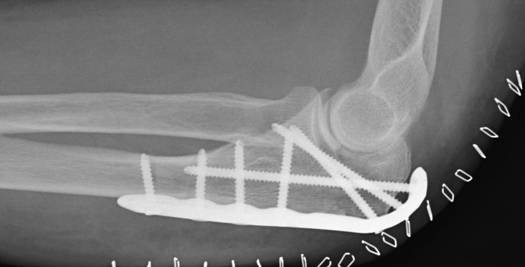
**The intermediate fracture fragment has been anatomically reduced into the trochlear notch**.

**Figure 14:**
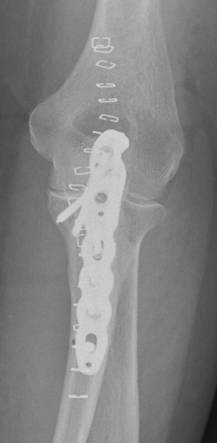
**Postoperative biplanar X-rays show situation after internal fixation**.

Reduction was verified by biplanar X-ray imaging. In order to restore the trochlear notch, temporary fixation of the intermediate fragment in anatomical position on the contour of the notch with respect to the distal aspect of the humerus was performed using K-wires and/or bone clamps [Figure 8]. For osteosynthesis a conventional plate contoured to the posterior surface of the ulna (standard, long proximal, 8-12 hole) or an pre-contoured locking plate was utilized [Figure 9]. One or two screws (2.7 mm) were inserted into the ulna shaft. An intramedullary 3.5 mm "home run" screw was inserted into the plate at the tip of the olecranon from proximal to distal into the ulna shaft. It was not advisable to utilize fixed angle screws in the ulna shaft prior to inserting the "home run" screw since the screws that are used to stabilize the plate to the ulna shaft may impair insertion of the intramedullary screw. Insertion of a distal screw into the ulna shaft using excentric drilling position exerted compression on the fracture and the trochlear notch. Additional interfragmentary screw fixation of single fragments may in single cases be necessary [Figure 10]. Insertion of the remaining shaft screws into the ulna completed the stable osteoynthesis [Figure 11]. A wound drainage (10 Charrière) was inserted, the wound was closed in layers, and a sterile wound dressing was applied [Figure 12]. A cast-splint was applied for three to four days during the initial wound healing phase. Careful postoperative treatment with active and active-assisted physiotherapy with range of motion limited by pain and discomfort was initiated. Postoperative X-ray control was performed after drainage removal [Figures 13, 14]. Clinical and radiological follow-up studies were performed in intervals after three, six and twelve weeks. The German version of the Patient-Rated Elbow Evaluation Score (PREE) includes a 20-item questionnaire designed to assess elbow pain and disability in activities of daily living and was calculated for 45 out of 80 patients at a minimum of 8 months postoperatively (range 8-84 months). Criteria of the PREE score include pain, function in specific activities and function in every day activities [18]. A total score out of 100 is computed by equally weighting the pain score (sum of five items) and the disability score (sum of fifteen items, divided by 3). No standard values for the total PREE score have been published yet [18]. Higher score indicates more pain and functional disability. In this study, a total score of 0 to 20 out of 100 points was considered to be an excellent result; 21 to 30 points, a good result; 31 to 40 points, a fair result; and >40 points, a poor result.

## Results

29 patients were treated with internal fixation with figure-of-eight tension band wire fixation and 51 patients with single posterior plate with and without intramedullary screw. 4 out of 29 patients with tension band wire fixation required revision surgery using stable plate fixation, and one patient with initial plate fixation underwent an operative revision [Table 1].

**Table 1:** Results after open reduction and internal fixation of complex olecranon fractures

***olecranon fractures (total)***	***ORIF: tension band wiring***	***ORIF: plate fixation***
80 patients	29/80 patients	51/80 patients
	***secondary revision:***	***secondary revision:***
	4/29 patients	1/51 patients

An intermediate fragment was seen in 52 patients in conventional radiography and/or CT scan [Table 2]. In 29 out of these 52 patients, the intermediate fragment was described in the operative report (whereas in 23 patients it was not). 24 of these 29 patients were treated with posterior plate osteosynthesis with or without an intramedullary screw, and five patients with figure-of-eight tension band wire fixation. Because of secondary dislocation, three of these five patients required operative revision, and the initial fixation with figure-of-eight tension band wire was replaced by posterior plate fixation and intramedullary screw. One patient required operative revision due to intraarticular position of one screw after posterior plate fixation. Complications of initial operative treatment were related to superficial infection (two patients), secondary dislocation followed by operative revision (three patients) and heterotopic ossifications (one patient) [Table 2].

**Table 2:** Results in patients with and without intermediate fragment

***intermediate fragment (IF)***	
52/80 patients	
***IF described in operative report:***	***IF not described in operative report:***
29/52 patients	23/52 patients
***ORIF: tension band wiring***	***ORIF: plate fixation***
5/29 patients	24/29 patients
***Complications:***	
Secondary dislocation: 3/5 patients	Intraarticular positioned screw: 1/24 patients
Superficial infection: 2/29 patients	
Heterotopic ossifications: 1/29 patients	

**Figure 15:**
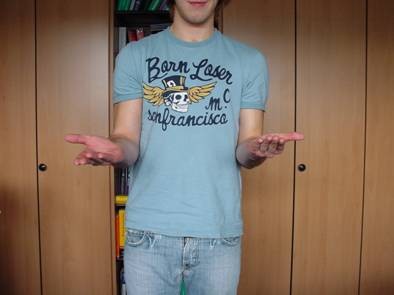
**Patient 1: Clinical results eight months after trauma were evaluated using the PREE score**.

**Figure 16:**
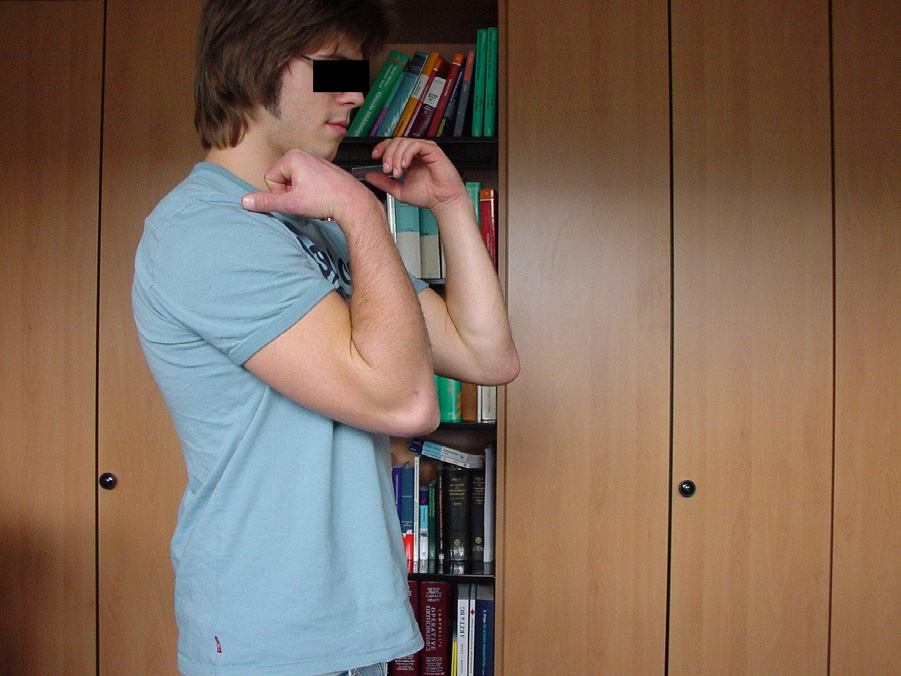
**Excellent clinical outcome**.

**Figure 17:**
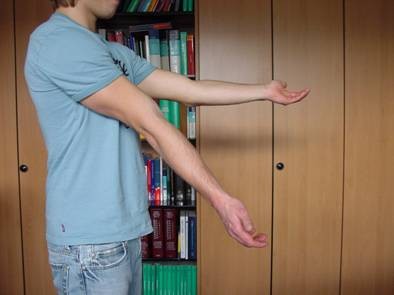
**0 out of 100 points in the PREE score**.

**Figure 18:**
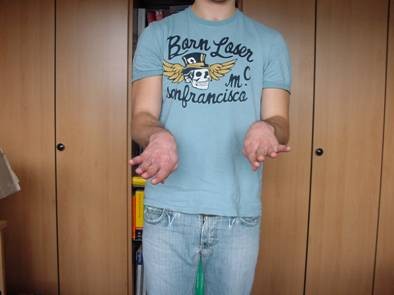
**Excellent functional result**.

Functional outcome using the PREE demonstrated a total score of 9 points (4 points for pain, 5 points for function in specific and daily activities, SD 0.9) on average in 45 out of 80 patients [Figures 15, 16, 17, 18]. 25 patients with posterior plate fixation with or without an intramedullary screw showed a total score of 8 points (SD 0.9), and 20 patients with figure-of-eight tension band wire fixation had a total score of 9 points (SD 0.6), on average.

## Discussion

**Figure 19:**
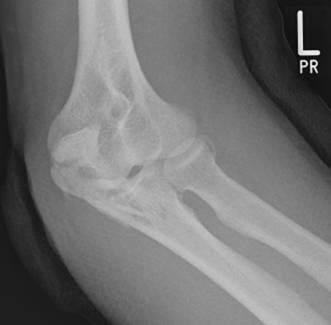
**Sometimes an intermediate fragment is not detectable in conventional biplanar X-rays**.

**Figure 20:**
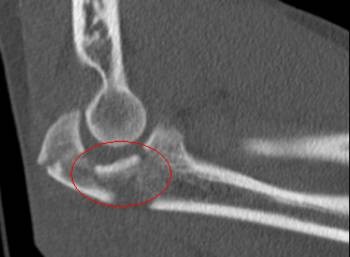
**Preoperative diagnostics should include CT imaging to detect an intermediate fragment**.

Fractures of the olecranon are surgically demanding due to the complexity of the elbow joint. Fracture-dislocations of the olecranon occur in anterior and posterior patterns with specific injury characteristics and pitfalls [19]. The therapeutic goal is to obtain a good and stable primary fixation as well as early active mobilization [20]. Primary principles of treatment are restoration of joint congruity and stability while permitting early range of motion [21]. Earlier studies point out the importance of anatomic reduction of the proximal ulna to restore the contour and dimensions of the trochlear notch of the ulna and to align the radiocapitellar joint [22-25]. It is well known that articular surface incongruity of more than 2 mm leads to posttraumatic arthritis [26]. These results motivated us to search for additional parameters to assess surgical treatment of olecranon fractures. We analyzed the fracture pattern and focused on identification of an intermediate fragment. In recent literature, there are several descriptions about so called key fragments in multi-fragmentary olecranon fractures, but these descriptions are unspecific. No conclusive reports have focused on detection, description, and specific surgical technique to stabilize the key fragment characterized as intermediate fragment, or have assessed its role in treatment and outcome of olecranon fractures. Despite its rare description in literature, the intermediate fracture fragment is commonly found in daily surgical practice. In approximately 2 out of 3 patients treated with the diagnosis of olecranon fracture an intermediate fragment was found. These significant results suggest that the pattern of olecranon fractures often includes an intermediate fracture fragment. The key to anatomic restoration of the trochlear notch of the olecranon and fracture reduction includes consideration, identification and anatomic reduction of the intermediate fragment in diagnostic work up and initial operative treatment. Accurate preoperative assessment of the olecranon fracture is very important: It may be possible to detect an intermediate fragment in biplanar radiographs, but sometimes it is not detectable in conventional X-rays [Figure 19]. Therefore, although CT scan is not mandatory, preoperative diagnostics should include CT imaging and careful assessment of the fracture pattern to detect an intermediate fragment [Figure 20].

In general, closed reduction techniques are not successful for anatomic reduction of the joint surface due to the entrapped intra-articular fracture fragments, especially the intermediate fragment. Various fixation techniques are utilized to restore the joint line and contour of the trochlear notch. Patients with tension band wiring require more frequently a second procedure for removal of symptomatic hardware than patients who underwent a plating procedure [12]. Whereas in olecranon fractures without intermediate fragment figure-of-eight tension band wiring remains presently the "golden standard", it is obsolete in complex olecranon fractures. For stable fixation of these fractures we favour plate osteosynthesis (conventional plate contoured to the posterior surface of the proximal ulna or alternatively pre-contoured locking plate) with an additional intramedullary "home run" screw. Compared with pre-contoured plates conventional plates are more prominent on the olecranon, often difficult to adapt to the bent end of the olecranon, and probably in many fracture patterns not strong enough. Therefore pre-contoured plates are favoured.

As in studies published previously, we did not find in this observation sufficient differences in outcome using the PREE score between patients with posterior plate osteosynthesis and patients with figure-of-eight tension band wire fixation. Data provided in our study do not support that olecranon plating is generally beneficial in complex olecranon fractures with intermediate fragment compared to tension band wiring. Nevertheless, we would announce that utilization of the "home run" screw secures reduction of fracture fragments including alignment of the intermediate fragment to the trochlear face. In the operative report precise description of the fracture pattern including presence of an intermediate fragment is recommended.

## Conclusions

Considering the pivotal role of the intermediate fragment in primary operative treatment of multi-fragmentary dislocated olecranon fractures we suggest to include the intermediate fragment into established classifications. In addition to existing classifications of olecranon fractures, the intermediate fragment should be recognized and mentioned separately.

Additional prospective studies with a long-time follow-up are necessary to assess and compare in a standardized way clinical and radiological outcome including detailed evaluation of the restored trochlear notch contour of the proximal ulna after primary operative treatment of olecranon fractures with intermediate fragment.

## Competing interests

The authors declare that they have no competing interests.

## Consent statement

Written informed consent was obtained from the patient for publication of this report and accompanying images. A copy of the written consent is available for review by the Editor-in-Chief of this journal.

## Authors' contributions

CVR, AW and CH contributed to conception and design of the study, acquisition of data, analysis and interpretation of data, and drafting the manuscript. OT and VB participated in design and coordination, helped to draft the manuscript and supervised the whole study. All authors read and approved the final manuscript.
